# Oxidative stress induced by the chemotherapeutic agent arsenic trioxide

**DOI:** 10.1007/s13205-013-0170-0

**Published:** 2013-09-13

**Authors:** Mathews V. Varghese, Alex Manju, M. Abhilash, M. V. Sauganth Paul, S. Abhilash, R. Harikumaran Nair

**Affiliations:** School of Biosciences, Mahatma Gandhi University, P.D Hills P.O, Kottayam, 686560 Kerala India

**Keywords:** Arsenic trioxide, Antioxidants, Lipid peroxidation, Glutathione, Oxidative stress

## Abstract

Arsenic compounds have been used for medicinal purposes throughout history. Arsenic trioxide (As_2_O_3_) achieved dramatic remissions in patients with acute promyelocytic leukaemia. Unfortunately, the clinical usefulness of As_2_O_3_ has been limited by its toxicity. The present study was designed to investigate the toxic effects of As_2_O_3_ at its clinical concentrations. Experimental rats were administered with As_2_O_3_ 2, 4 and 8 mg/kg body weight for a period of 45 days and the serum glucose, creatine kinase, lactate dehydrogenase, lipid peroxidation and antioxidant status were measured. As_2_O_3_-treated rats showed elevated serum glucose, creatine kinase and lactate dehydrogenase concentrations. Lipid peroxidation product malondialdehyde was found to be produced more in arsenic-treated rats. Reduced glutathione and glutathione-dependant antioxidant enzymes, glutathione-*S*-transferase and glutathione peroxidase, and the antiperoxidative enzymes, superoxide dismutase and catalase, concentrations were reduced with the As_2_O_3_ treatment. All these toxic effects were found increased with the increase in concentration of As_2_O_3_. The results of the study indicate that As_2_O_3_ produced dose-dependant toxic side effects at its clinical concentrations.

## Introduction

Arsenic is widely distributed in air, water and soil in the form of either metalloids or chemical compound (Basu et al. 2001). The most toxicologically potent arsenic compounds are in the trivalent oxidation state (Hughes 2002). Exposure to arsenic occurs via ingestion, inhalation, dermal contact, and the parenteral route (Tchounwou et al. 1999). The health effects that are associated with arsenic exposure include cardiovascular and peripheral vascular disease, developmental anomalies, neurologic and neurobehavioral disorders, diabetes, portal fibrosis, and multiple cancers (Tseng et al. 2003). Haematological abnormalities associated with arsenic intoxication are haemoglobinuria, intravascular coagulation, bone marrow depression, severe pancytopenia, and normocytic normochromic anaemia and basophilic stippling (Ratnaike 2003).

The treatment with low doses of arsenic trioxide (As_2_O_3_) caused high rates of complete remission in patients suffering from acute promyelocytic leukaemia (APL) (Wang and Chen 2008). The adverse effects associated with As_2_O_3_ treatment include hyperleukocytosis, APL differentiation syndrome, electrocardiographic abnormalities (QTc-interval prolongation), peripheral neuropathy, skin rash, gastrointestinal reactions and hyperglycaemia (Rust and Soignet 2001). Arsenic compounds also showed genotoxic effects; induced gene amplification, inhibit DNA repair, and induce expression of the oxidative stress protein heme oxygenase in mammalian cells (Vega et al. 1995; Keyse et al. 1990).

Due to the toxic side effects of arsenic, it carries significant risks in their therapeutic regiment. The mechanisms of arsenic-induced toxic effects during clinical trials were not fully elucidated. So the present study was performed to understand the toxicological association between biochemical parameters and the blood antioxidant status at different clinically relevant concentrations of As_2_O_3_.

## Materials and methods

### Chemicals

Arsenic trioxide, sodium pyruvate, thiobarbituric acid and triton X-100, phenazine methosulphate, nitroblue tetrazolium were obtained from Sigma-Aldrich (Bangalore, India). l-aspartate, α-oxoglutarate, 2,4-dinitro phenyl hydrazine, nicotinamide adenine dinucleotide (reduced), 1-chloro-2,4-dinitrobenzene (CDNB), 5,5′-dithiobis-(2-nitrobenzoic acid), nicotinamide adenine dinucleotide phosphate and reduced glutathione were purchased from Merck Specialties Pvt Ltd (Mumbai, India). All other chemicals were purchased from Sisco Research Laboratories (Mumbai, Maharashtra, India).

### Experimental animals

Male albino rats of Wistar strain (200–220 g) were purchased from Small Animal Breeding Station of Government Veterinary College, Mannuthy, Thrissur, Kerala, India. The animals were housed in polypropylene cages kept in the animal house of School of Biosciences, Mahatma Gandhi University, Kottayam. All the animals were maintained under standard laboratory conditions of temperature (22 ± 3 °C) and 12-h light and dark cycles throughout the experimental period. Rats were provided with laboratory chow (Hindustan Unilever Ltd., Mumbai, India) and water ad libitum. Experiments were conducted as per the guidelines of Institutional Animal Ethical Committee, School of Biosciences, Mahatma Gandhi University (Reg. No. B1662009/2). After 2 weeks of acclimation, animals were randomly divided into four groups with six animals in each group.

- Group 1: Normal Control
- Group 2: As_2_O_3_ 2 mg/kg b.wt
- Group 3: As_2_O_3_ 4 mg/kg b.wt
- Group 4: As_2_O_3_ 8 mg/kg b.wt

The duration of the study was 45 days and the route of administration was daily by oral intubation. At the end of the experimental period, blood was drawn from the orbital sinus of the rat’s eye. Anticoagulated blood was used for the analysis of blood antioxidant status and lipid peroxidation. The blood samples collected in another set of test tubes without anticoagulant were centrifuged at 3,000 rpm at 4 °C for 20 min; the clear serum obtained was used for the biochemical assays.

### Analysis of serum glucose, creatine kinase and lactate dehydrogenase

Serum Glucose, Creatine Kinase (CK) and Lactate dehydrogenase (LDH) were detected (Agappe Diagnostic Ltd., Ernakulam, Kerala, India) using semi-auto analyzer (RMS, India).

### Analysis of blood antioxidant status and lipid peroxidation

Haemoglobin concentration in blood was determined according to the method of Drabkin and Austin (1932). Superoxide dismutase (SOD) activity was determined by the method of Paoletti et al. (1986). Catalase (CAT) activity was measured in the sample according to the method of Aebi (1984) by measuring the decrease in absorbance of hydrogen peroxide (H_2_O_2_) at 240 nm. Glutathione-*S*-Transferase (GST) activity was estimated by determining the rate of formation of glutathione and CDNB conjugates (Beutler et al. 1986). Glutathione peroxidase (GPx) activity was measured by the method of Paglia and Valentine (1967). Reduced glutathione (GSH) was estimated by the method of Beutler et al. (1963). Malondialdehyde (MDA), a product of lipid peroxidation, was determined by the method of Buege and Aust (1978).

### Statistical analysis

One-way ANOVA followed by LSD post hoc multiple comparison test was used for comparison among the four groups (SPSS/PC+ version 18, SPSS Inc. Chicago, Illinois, USA). Probability (*p*) <0.05 was considered statistically significant.

## Results

### As_2_O_3_-induced alterations in the serum Glucose, CK and LDH

**Fig. 1:**
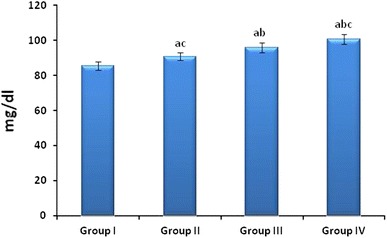
Effect of As_2_O_3_ on Serum Glucose: Normal control (Group I), As_2_O_3_ 2 mg/kg b.wt (Group II), As_2_O_3_ 4 mg/kg b.wt (Group III), and As_2_O_3_ 8 mg/kg b.wt (Group IV). Data represented as mean ± SD, *n* = 6. *p* < 0.05 was considered significant. ^a^Statistical significance in comparison to normal control, ^b^statistical significance in comparison to group II, and ^c^statistical significance in comparison to group III

**Fig. 2:**
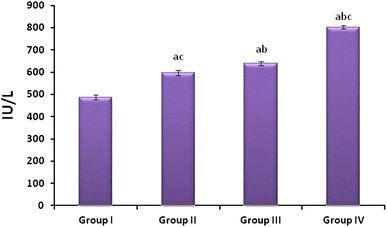
Effect of As_2_O_3_ on Creatine Kinase: Normal control (Group I), As_2_O_3_ 2 mg/kg b.wt (Group II), As_2_O_3_ 4 mg/kg b.wt (Group III), and As_2_O_3_ 8 mg/kg b.wt (Group IV). Data represented as mean ± SD, *n* = 6. *p* < 0.05 was considered significant. *p* < 0.05 was considered significant. ^a^Statistical significance in comparison to normal control, ^b^statistical significance in comparison to group II, and ^c^statistical significance in comparison to group III

**Fig. 3:**
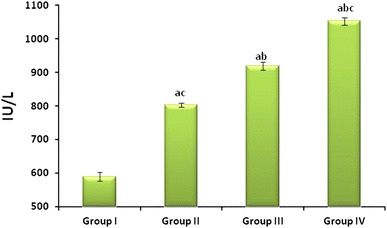
Effect of As_2_O_3_ on Lactate Dehydrogenase: Normal control (Group I), As_2_O_3_ 2 mg/kg b.wt (Group II), As_2_O_3_ 4 mg/kg b.wt (Group III), and As_2_O_3_ 8 mg/kg b.wt (Group IV). Data represented as mean ± SD, *n* = 6. *p* < 0.05 was considered significant. ^a^Statistical significance in comparison to normal control, ^b^statistical significance in comparison to group II, and ^c^statistical significance in comparison to group III

As_2_O_3_ treatment significantly (*p* < 0.05) increased the serum Glucose (Fig. 1), CK (Fig. 2) and LDH (Fig. 3) when compared to the group I control. These biochemical parameters also showed statistical significance (*p* < 0.05) when compared between groups II, III and IV. The concentrations of Glucose, CK and LDH were increased with respect to the increase in concentration of As_2_O_3_.

### As_2_O_3_-induced lipid peroxidation

The lipid peroxidation product MDA was significantly increased with As_2_O_3_ treatment when compared to the group I control. In statistical analysis, the intergroup comparison between As_2_O_3_-treated groups II, III and IV showed significant variation with respect to the increase in dose of As_2_O_3_ (Table 1).

**Table 1:** Effect of As_2_O_3_ on the Blood Antioxidant Status: Normal control (Group I), As_2_O_3_ 2 mg/kg b.wt (Group II), As_2_O_3_ 4 mg/kg b.wt (Group III), and As_2_O_3_ 8 mg/kg b.wt (Group IV)

Parameters	Group I	Group II	Group III	Group IV
MDA (μM/L)	4.09 ± 0.12	4.68 ± 0.17^ac^	4.9 ± 0.09^ab^	5.05 ± 0.11^abc^
GSH (μM/gHb)	5.87 ± 0.32	5.16 ± 0.2^ac^	4.47 ± 0.32^ab^	3.84 ± 0.42^abc^
SOD (U/mgHb)	1.59 ± 0.13	1.32 ± 0.12^ac^	0.98 ± 0.14^ab^	0.75 ± 0.17^abc^
CAT (k/ml)	10.31 ± 0.45	9.12 ± 0.85^ac^	8.28 ± 0.65^ab^	7.44 ± 0.47^abc^
GPx (U/gHb)	7.94 ± 0.18	7.44 ± 0.11^ac^	7.12 ± 0.12^ab^	6.16 ± 0.1^abc^
GST (μM/min/gHb)	2.09 ± 0.11	1.64 ± 0.09^ac^	1.35 ± 0.09^ab^	0.76 ± 0.07^abc^

Data represented as mean ± SD, *n* = 6

*p* < 0.05 was considered significant

^a^Statistical significance in comparison to normal control

^b^Statistical significance in comparison to group II

^c^Statistical significance in comparison to group III

### As_2_O_3_-induced alterations in the antioxidant status

The tripeptide GSH was reduced significantly with respect to the control and was noticed in the arsenic-treated groups. As_2_O_3_ treatment also decreased the GSH-dependant antioxidant enzymes, GST and GPx, and the antiperoxidative enzymes, SOD and CAT, when compared to the group I control and between the arsenic-treated groups II, III and IV. These reductions in the antioxidant enzyme activities were in accordance with the increase concentration of As_2_O_3_ (Table 1).

## Discussion

As_2_O_3_ is an effective cancer therapeutic drug for acute promyelocytic leukaemia and has potential anticancer activity against a wide range of solid tumours (Lu et al. 2007). The adverse effects that have been noted in clinical trials of As_2_O_3_ are fluid retention, cardiac toxicity, hepatocellular toxicity and electrocardiographic changes (Soignet et al. 2001). The duration of the present study was selected based on the previous clinical study (Huan et al. 2000). They have reported that As_2_O_3_ treatment resulted in significant remission in APL patients. The As_2_O_3_ concentrations in our study are in the range of clinically available concentrations for anti-leukaemia treatment (Li et al. 2002).

In the present study, the treatment with As_2_O_3_ increased the glucose concentration in serum. Miller et al. (2002) reported that trivalent arsenic inhibits the uptake of glucose into cells, gluconeogenesis, fatty acid oxidation and further production of acetyl CoA. Pyruvate dehydrogenase, an enzyme of glucose metabolism, is susceptible to arsenic-induced reactive oxygen species (ROS) generation (Aposhian and Aposhian 2006). The thiol moiety is an important target for arsenic (Flora 2011). The increased concentration of glucose in this study, may be due to the binding of arsenic to the sulfhydryl groups of glucose metabolising enzymes, and thereby blocked the uptake of glucose. The altered blood sugar level may also due to islet cells toxicity, because arsenic administration caused severe pancreatic damage (Mukherjee et al. 2004). In our observation, the treatment with As_2_O_3_-induced ROS production, which may reduce insulin production by pancreatic cellular damage, leads to the increased glucose concentration in serum.

As_2_O_3_ administration caused myocardial damage and increased release of CK and LDH in serum (Raghu et al. 2009). In our recent report, the As_2_O_3_ treatment caused oxidative stress and structural aberrations in the cardiac tissue of experimental rats (Mathews et al. 2013). So the increased concentration of CK and LDH observed in this investigation may be due to the exudation of enzymes from cells to the systemic circulation because of cellular damage induced by the As_2_O_3_.

From our observation, it is found that the MDA production in blood is significantly increased with As_2_O_3_ treatment. MDA is a marker of endogenous lipid peroxidation. Liu et al. (2001) reported that the treatment with arsenic caused a significant increase in the rate of formation of ROS such as superoxide anion radical, hydroxyl radical and hydrogen peroxide. The toxic potential exerted by these compounds is through their reactivity with sulphur containing compounds and the generation of ROS (Hughes et al. 2011). Arsenic-induced MDA production could be due to the impairment of cells’ natural protective system and could be directly related to the GSH depletion in blood cells (Wang et al. 2006). Higher the rate of MDA production corresponds to As_2_O_3_ inversely associated with GSH. Depletion of GSH results in the increased production of arsenic-induced ROS, which may enhance the lipid peroxidation as observed in the present study.

The most significant alteration in the antioxidant defence is the decrease in GSH concentration, and GSH has direct antioxidant activity (Schulz et al. 2000). In this study, As_2_O_3_-treated rats showed decreased concentration of GSH and GSH-dependant antioxidant enzymes GPx and GST. This reduction is suggested to be due to the consumption of glutathione while protecting against the arsenic-induced oxidative stress, for maintaining cellular redox status (Hughes 2002). As_2_O_3_ administration reduced the antioxidant and antiperoxidative enzyme concentration in liver tissue of experimental rats (Mathews et al. 2012a). GPx and GST play an important role in arsenic detoxification and the arsenic-induced oxidative stress (Thompson et al. 2009). GST utilises GSH as a cofactor, and therefore, the decrease in the activity of GST after As_2_O_3_ treatment may suggest coming from the paucity of GSH.

The exposure to arsenic decreased the activities of antiperoxidative enzymes SOD and CAT. The decreased SOD activity in serum suggested that the accumulation of superoxide anion radical might be responsible for increased lipid peroxidation following arsenic treatment (Maiti and Chatterjee 2000). ROS can themselves reduce the activity of the antioxidant enzymes CAT and GPx (Datta et al. 2000). Reduction in the antioxidant and antiperoxidative enzymes during As_2_O_3_ treatment may leads to the deposition of arsenic in tissues (Mathews et al. 2012b). SOD catalyses the dismutation of superoxide anions and prevents the subsequent formation of hydroxyl radicals in blood cells (Wang et al. 2006). In the present study, the decreased SOD activity may suggest that the accumulation of superoxide anion radical; might be responsible for increased lipid peroxidation following arsenic treatment as observed by Maiti and Chatterjee (2000). Exposure to arsenic decreased the CAT activity. CAT catalyses the removal of H_2_O_2_ formed during the reaction catalysed by SOD (Lee and Ho 1995). In the present study, the decreased CAT activity indicates the impaired ability to detoxify H_2_O_2_ and may leads to the accumulation of H_2_O_2_ and thereby oxidative stress.

## Conclusion

In the current investigation, As_2_O_3_ treatment at its clinically different concentrations induced toxic effects by varying the blood glucose, CK, LDH and the oxidative status. As_2_O_3_-induced oxidative stress and the lipid peroxidation may be due to the reduced activity of GSH and GSH-dependant antioxidant and antiperoxidative enzymes. We also suggest that further studies are necessary for identifying the cellular and molecular mechanism of toxicity of arsenic at its clinical concentrations.
